# Palliative primary tumor resection may not offer survival benefits for patients with unresectable metastatic colorectal neuroendocrine neoplasms, one multicenter retrospective cohort study

**DOI:** 10.1186/s12893-024-02380-9

**Published:** 2024-03-12

**Authors:** Guozhi Yu, Shen Liu, Zhijie Wang, Qian Liu, Hongchang Ren, Wenhui Hu

**Affiliations:** 1 Department of Colorectal and Anal Surgery, Beijing Erlonglu Hospital, Beijing, 100016 China; 2 Department of Colorectal Surgery, National Clinical Research Center for Cancer/Cancer Hospital, National Cancer Center, Chinese Academy of Medical Sciences and Peking Union Medical College, No. 17, Panjiayuan Nanli, Chaoyang District, Beijing, 100021 China; 3 Key Laboratory of Carcinogenesis and Translational Research (Ministry of Education/Beijing), Gastrointestinal Cancer Center, Peking University Cancer Hospital & Institute, Beijing, 100142 China; 4 Department of General Surgery, Strategic Support Force Medical Center, No.9, Anxiang North, Desheng Gate, Chaoyang District, Beijing, 100101 China

**Keywords:** Colon, Rectum, Neuroendocrine, Neoplasms, Surgery

## Abstract

**Background:**

The efficacy of palliative primary tumor resection (PTR) in improving prognosis for patients with unresectable metastatic colorectal neuroendocrine neoplasms (NENs) has not been fully explored.

**Methods:**

We performed one retrospective cohort study and recruited 68 patients with unresectable metastatic colorectal NENs from two Chinese medical centers between 2000 and 2022. All patients were assigned to PTR group and no PTR group. The clinicopathological manifestation data were carefully collected, and the survival outcomes were compared between the two groups using Kaplan–Meier methods. Propensity score matching (PSM) was conducted to minimize confounding bias. Univariate and multivariate Cox proportional hazards regression analyses were performed to identify prognostic factors.

**Results:**

A total of 32 patients received PTR, and the other 36 patients did not. The median progression-free survival (PFS) and overall survival (OS) times were 4 and 22 months in the whole cohort, respectively. For patients who received no PTR, the median OS was 16 months, and the 1-year OS rate and 3-year OS rate were 56.4% and 39.6%, respectively. For patients who received PTR, the median OS was 24 months, and the 1-year OS rate and 3-year OS rate were 67.9% and 34.1%, respectively. However, the Kaplan–Meier survival curves and log-rank test demonstrated no significant survival difference between the two groups (*P* = 0.963). Moreover, palliative PTR was also not confirmed as a prognostic factor in subsequent univariable and multivariable Cox proportional hazards regression analyses in both the original and matched cohorts. Only histological differentiation was identified as an independent prognostic factor affecting PFS [hazard ratio (HR) = 1.86, 95% confidence interval (CI): 1.02–3.41, *P* = 0.043] and OS [HR = 3.70, 95% CI: 1.09–12.48, *P* = 0.035] in the original cohort.

**Conclusions:**

Palliative PTR may not offer survival benefits for patients with unresectable metastatic colorectal NENs.

## Introduction

Colorectal neuroendocrine neoplasms (NENs) are rare diseases arising from the diffuse neuroendocrine system of the colon and rectum. Although colorectal NENs constitute less than 1% of all colorectal tumors, a rapid increase in their incidence has been observed in recent years, owing to the popularization of colorectal cancer coloscopy programs [1, 2]. Colorectal NENs are a heterogeneous group of diseases with varying clinical manifestations and malignant potentials, ranging from diminutive, indolent, and early NENs with favorable prognosis to huge, highly aggressive, and metastatic NENs with dismal oncological outcomes that depend on their histological grade and differentiation [3, 4].

The incidence rate of distant metastasis was approximately 11–14% for all colorectal NENs at initial diagnosis [5, 6]. However, the probability of metastatic disease varies widely from G1 to G3 NENs. For G1 and G2 NENs, they were only 0.3% and 6.3%, respectively [7]. However, for G3 NENs, more than half can have distant metastasis at diagnosis [8, 9]. Tumor grade, histological differentiation, depth of tumor invasion, and size were risk factors for distant metastasis [10, 11]. The liver is the most common organ of metastases, followed by distant lymph nodes, peritoneum, lung, and bone [8, 9]. More than half of metastatic patients show multiple metastases that cannot be resected radically [12].

Numerous previous studies have explored the efficacy of primary tumor resection (PTR) in prolonging survival for unresectable colorectal adenocarcinomas, leading to controversial conclusions. Most retrospective studies have suggested that PTR may offer survival benefits in carefully selected patients [13–15]. However, a randomized clinical trial from Japan showed that PTR followed by chemotherapy did not offer any survival benefit over chemotherapy alone [16]. However, there is a paucity of data on the therapeutic effects of PTR for the management of unresectable metastatic colorectal NENs, and optimal therapeutic strategies have not been well established for these patients [17]. This retrospective cohort study aims to explore whether PTR can offer survival benefits for patients with uncurable metastatic colorectal NENs.

## Methods

### Patients

Our study received approval from the ethics committee of the National Cancer Center and followed the rules of the Helsinki Declaration of the World Medical Association. Informed consent was obtained from all participants. A total of 68 consecutive patients were included between 2000 and 2022, with 60 from the National Cancer Center/Cancer Hospital, Chinese Academy of Medical Sciences, and the other 8 from the Strategic Support Force Medical Center. All patients were histologically diagnosed with colorectal NENs through pathological reviews and immunohistochemical examinations. All patients were definitely diagnosed with metastatic NENs that could not be resected with curative intent through imaging examinations at the initial date of diagnosis. We designed a retrospective cohort study; 32 patients received palliative resection of primary colorectal NENs, and the other 36 patients did not undergo palliative surgery. Data including demographic information, clinicopathological features, and survival outcomes were obtained through medical records and telephone calls. The last date of follow-up was July 30, 2023. The primary outcomes of interest were progression-free survival (PFS) time and overall survival (OS) time. The PFS was calculated between the data of initial diagnosis and cancer progression confirmed by imaging evaluation. OS was obtained between the initial diagnosis and cancer-specific mortality data.

### Statistical analysis

All statistics were performed using the Statistical Package for the Social Sciences (SPSS version 24.0, IBM Corp., Armonk, NY, United States). Quantitative data are presented as the means ± standard deviations (SD) and were compared using t tests if they followed a normal distribution. Quantitative data that did not follow the normal distribution are shown as medians and ranges and were compared through Mann–Whitney U tests. Qualitative and ordinal data were described as frequencies with percentages and were compared through χ^2^ tests for qualitative variables and Mann–Whitney U tests for ordinal variables. Propensity score matching (PSM) was performed by fitting a logistic regression model and setting the caliper at 0.1. One-to-one pair matching was performed without replacement, and 26 matched pairs were selected. The covariates included gender, age, Eastern Cooperative Oncology Group (ECOG) score, the primary tumor location, grade, differentiation, TNM T stage, TNM N stage, site of metastases, cycles of chemotherapy. PFS and OS rates were calculated from the Kaplan–Meier survival curves and compared using the log-rank test. Univariable and multivariable Cox proportional hazards regression analyses were performed to identify the independent risk factors affecting clinical outcomes.

## Results

### Patient characteristics

Between 2000 and 2022, a total of 68 consecutive patients with unresectable metastatic colorectal NENs were included in our study. The data regarding the demographics and clinicopathological manifestations are detailed in Table 1. Our study included 40 (58.8%) males and 28 (41.2%) females, with a mean age of 57.8 ± 14.2 years. Ten (14.7%) patients were fully functional (ECOG score = 0), 46 (67.6%) had an ECOG 1 score, 11 (16.2%) had an ECOG 2 score and 1 (1.5%) had an ECOG 3 score. Most (69.1%) patients had their primary tumor located in the rectum, followed by the cecum and ascending colon (17.6%) and sigmoid colon (8.8%). Five (7.4%), 15 (22.1%), and 48 (70.6%) had G1, G2, and G3 NENs, respectively. With regard to histological differentiation, 27 (39.7%) and 41 (60.3%) had well-differentiated neuroendocrine tumors (NETs) and poorly differentiated neuroendocrine carcinomas (NECs), respectively. The liver was the most common site of distant metastasis (79.4%), followed by distant lymph nodes (23.5%) and bone (17.6%). Thirty-two patients received palliative resection of the primary colorectal NENs, including Dixon (*n* = 12), Miles (*n* = 3), Hartmann (*n* = 1), sigmoidectomy (*n* = 5), right hemicolectomy (*n* = 11). The major reason for palliative resection was reduction of the tumor burden in asymptomatic patients (11, 34.4%) and relief of obstruction (14, 43.8%) and bleeding (7, 21.9%) in symptomatic patients. Among patients who did not undergo palliative resection of the primary colorectal NENs, 5 received colostomy, and 1 received intestinal stent placement due to bowel obstruction. As for the first-line chemotherapy regimen, 27 patients received platinum-based chemotherapy, and 22 patients received fluorouracil-based chemotherapy. The median number of chemotherapy cycles was 5 (range: 0–42 cycles).

**Table 1:** Demographic and clinicopathological manifestations

Variables	All (*n* = 68)
Gender, *n* (%)	
Male	40 (58.8%)
Female	28 (41.2%)
Age (yr, mean ± SD)	57.8 ± 14.2
ECOG score, *n* (%)	
0	10 (14.7%)
1	46 (67.6%)
2	11 (16.2%)
3	1 (1.5%)
4	0
5	0
Primary tumor site, *n* (%)	
Rectum	47 (69.1%)
Sigmoid colon	6 (8.8%)
Descending colon	1 (1.5%)
Transverse colon	2 (2.9%)
Cecum and ascending colon	12 (17.6%)
Grade	
G1	5 (7.4%)
G2	15 (22.1%)
G3	48 (70.6%)
Differentiation	
NET	27 (39.7%)
NEC	41 (60.3%)
Synaptophysin, *n* (%)	
Positive	61 (94.1%)
Negative	1 (1.5%)
Unknown	3 (4.4%)
Chromogranin, *n* (%)	
Positive	39 (57.4%)
Negative	23 (33.8%)
Unknown	6 (8.8%)
CD56, *n* (%)	
Positive	50 (73.5%)
Negative	6 (8.8%)
Unknown	12 (17.6%)
Ki67 [median (range)]	50% (1%, 95%)
Clinical T stage, *n* (%)	
T1, T2	6 (8.8%)
T3, T4	62 (91.2%)
Clinical N stage, *n* (%)	
N0	8 (11.8%)
N1	60 (90.2%)
Site of metastasis, *n* (%)	
Liver	54 (79.4%)
Lung	2 (2.9%)
Bone	12 (17.6%)
Distant lymph nodes	16 (23.5%)
Peritoneum	5 (7.4%)
Others	3 (4.4%)
Treatment strategy	
PTR + chemotherapy	13 (19.1%)
Chemotherapy + PTR	4 (5.9%)
Chemotherapy + PTR + chemotherapy	6 (8.8%)
Chemotherapy alone	32 (47.1%)
PTR alone	9 (13.2%)
No treatment	4 (5.9%)
Operation methods, *n* (%)	
Dixon	12 (17.6%)
Miles	3 (4.4%)
Hartmann	1 (1.5%)
Sigmoidectomy	5 (7.4%)
Right hemicolectomy	11 (16.2%)6
Only colostomy	5 (7.4%)
Only stent placement	1 (1.5%)
First-line chemotherapy regimen	
Fluorouracil-based regimens	22 (32.3%)
Platinum-based regimens	27 (39.7%)
Anlotinib	3 (4.4%)
No chemotherapy	13 (11.8%)
Unknown	5 (11.8%)
Cycles of chemotherapy [median (range)]	5 (0, 42)

SD: standard deviation, ECOG: Eastern Cooperative Oncology Group, NET: neuroendocrine tumor, NEC: neuroendocrine carcinoma, PTR: primary tumor resection

The differences in the clinicopathological variables between patients received PTR and those who did not receive PTR before and after PSM are presented in Table 2. Statistical analysis demonstrated that the two groups were comparable with regard to the demographics and clinicopathological variables in both the original cohort and the matched cohort.

**Table 2:** Clinicopathological manifestations between patients who received PTR and those who did not before and after PSM

Variables	Original cohort			Matched cohort		
	PTR (*n* = 32)	No PTR (*n* = 36)	*P*	PTR (*n* = 26)	No PTR (*n* = 26)	*P*
Gender, *n* (%)			0.931			1
Male	19 (59.4%)	21 (58.3%)		14 (53.8%)	14 (53.8%)	
Female	13 (40.6%)	15 (41.7%)		12 (46.2%)	12 (46.2%)	
Age (yr, mean ± SD)	58.5 ± 9.8	57.3 ± 14.5	0.696	57.8 ± 8.7	57.5 ± 14.7	0.798
ECOG score			0.092			1
0–1	29 (90.6%)	27 (75%)		23 (88.5%)	23 (88.5%)	
≥ 2	3 (9.4%)	9 (25%)		3 (11.5%)	3 (11.5%)	
Primary tumor site, *n* (%)			0.916			0.760
Rectum	22 (68.8%)	25 (69.4%)		19 (73.1%)	18 (69.2%)	
Colon	3 (9.4%)	3 (8.3%)		7 (26.9%)	8 (30.8%)	
Grade			0.676			0.506
G1	3 (9.4%)	2 (5.6%)		3 (11.5%)	2 (7.7%)	
G2	8 (25%)	7 (19.4%)		8 (30.8%)	5 (19.2%)	
G3	21 (65.6%)	27 (75%)		15 (57.7%)	19 (73.1%)	
Differentiation			0.102			0.262
NET	16 (50%)	11 (30.6%)		13 (50%)	9 (34.6%)	
NEC	16 (50%)	25 (69.4%)		13 (50%)	17 (65.4%)	
Clinical T stage, *n* (%)			0.562			0.638
T1, T2	4 (12.5%)	2 (5.6%)		3 (11.5%)	2 (7.7%)	
T3, T4	28 (87.5%)	34 (94.4%)		23 (88.5%)	24 (92.3%)	
Clinical N stage, *n* (%)			0.340			1
N0	2 (6.3%)	6 (16.7%)		1 (3.8%)	1 (3.8%)	
N1	30 (93.8%)	30 (83.3%)		25 (96.2%)	25 (96.2%)	
Site of metastasis, *n* (%)			0.457			0.781
Single-organ	18 (56.3%)	17 (47.2%)		13 (50%)	12 (46.2%)	
Multiple-organ	14 (43.8%)	19 (52.8%)		13 (50%)	14 (53.8%)	
Cycles of chemotherapy [median (range)]	5 (0, 42)	5 (0, 22)	0.848	5 (0, 33)	6 (0, 22)	0.392

PTR: primary tumor resection, PSM: propensity score matching, SD: standard deviation, ECOG: Eastern Cooperative Oncology Group, NET: neuroendocrine tumor, NEC: neuroendocrine carcinoma

### Survival outcomes

**Fig. 1:**
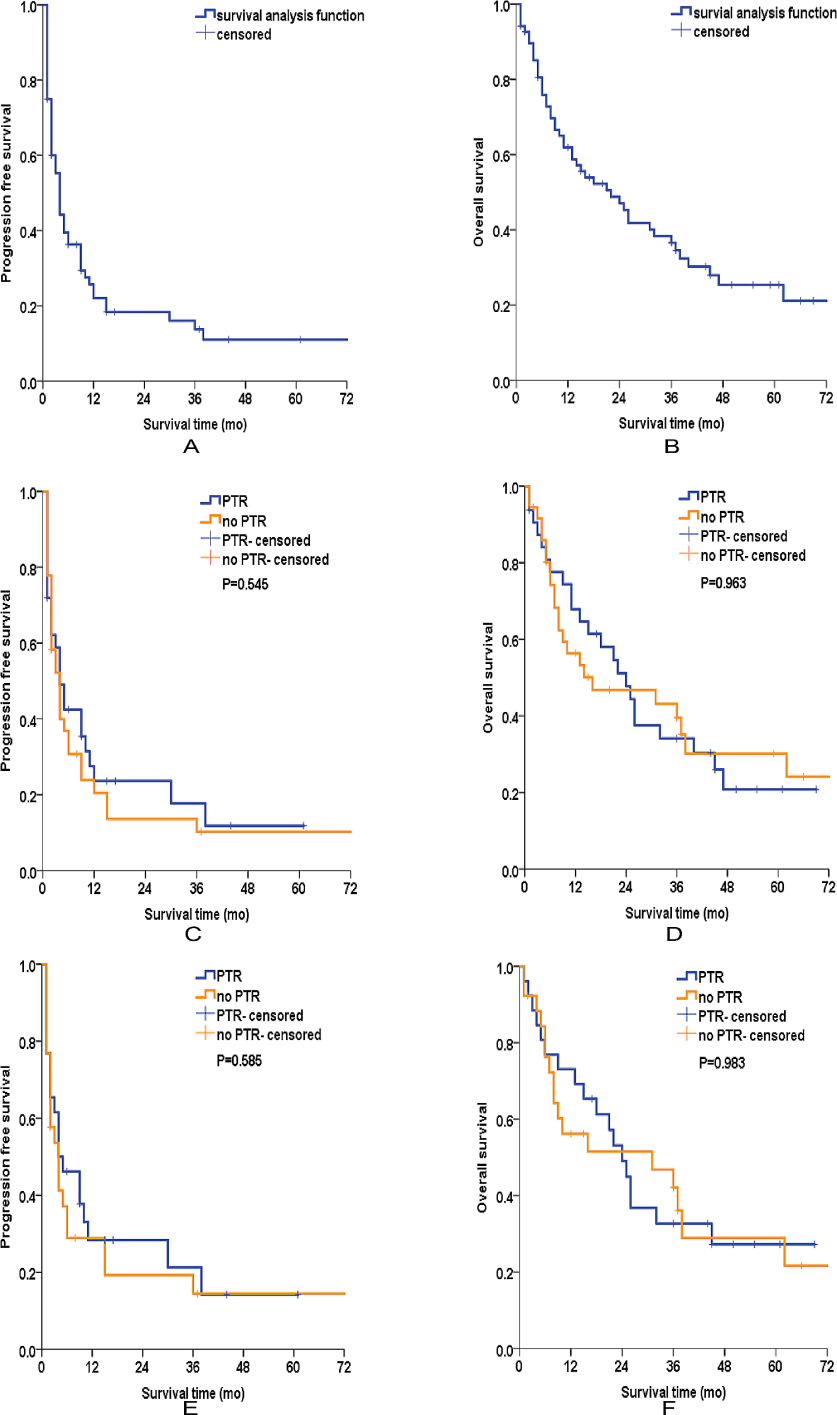
Kaplan–Meier survival analyses of patients before and after PSM. (**A**) PFS of the whole cohort, (**B**) OS of the whole cohort, (**C**) PFS of patients who received PTR and those who did not in the original cohort, (**D**) OS of patients who received PTR and those who did not in the original cohort, (**E**) PFS of patients who received PTR and those who did not in the matched cohort, (**F**) OS of patients who received PTR and those who did not in the matched cohort. PTR: primary tumor resection, PSM: propensity score matching, PFS: progression free survival, OS: overall survival

A median follow-up of 15.5 months (range 1–96 months) was achieved in our study. Of the 68 cases, 6 were lost to follow-up due to loss of communication or unexpected death from other accidents, resulting in a follow-up rate of 91.2%. In the entire cohort, the median PFS was 4 months, and the 1-year PFS rate and 3-year PFS rate were 25.7% and 13.8% in the cohort, respectively (Fig. 1A). With regard to OS, the median OS was 22 months, and the 1-year OS rate and 3-year OS rate were 61.9% and 36.6%, respectively (Fig. 1B). For patients who received PTR, the median PFS was 4 months, with 1-year and 3-year PFS rates of 23.6% and 17.7%, respectively. The median OS was 24 months, with 1-year and 3-year OS rates of 67.9% and 34.1%, respectively. For patients who did not receive PTR, the median PFS were also 4 months, with 1-year and 3-year PFS rates of 20.5% and 10.2%, respectively. The median OS was 16 months, with 1-year and 3-year OS rates of 56.4% and 39.6%, respectively. The Kaplan–Meier survival curves showed that there was no significant difference in terms of PFS (*P* = 0.545) and OS (*P* = 0.963) between the patients who underwent PTR and those who did not (Fig. 1C and D). Kaplan–Meier survival curves were then plotted in the matched cohort, and no survival benefit for PFS (*P* = 0.585) and OS (*P* = 0.983) was observed for patients who received PTR (Fig. 1E and F).

**Fig. 2:**
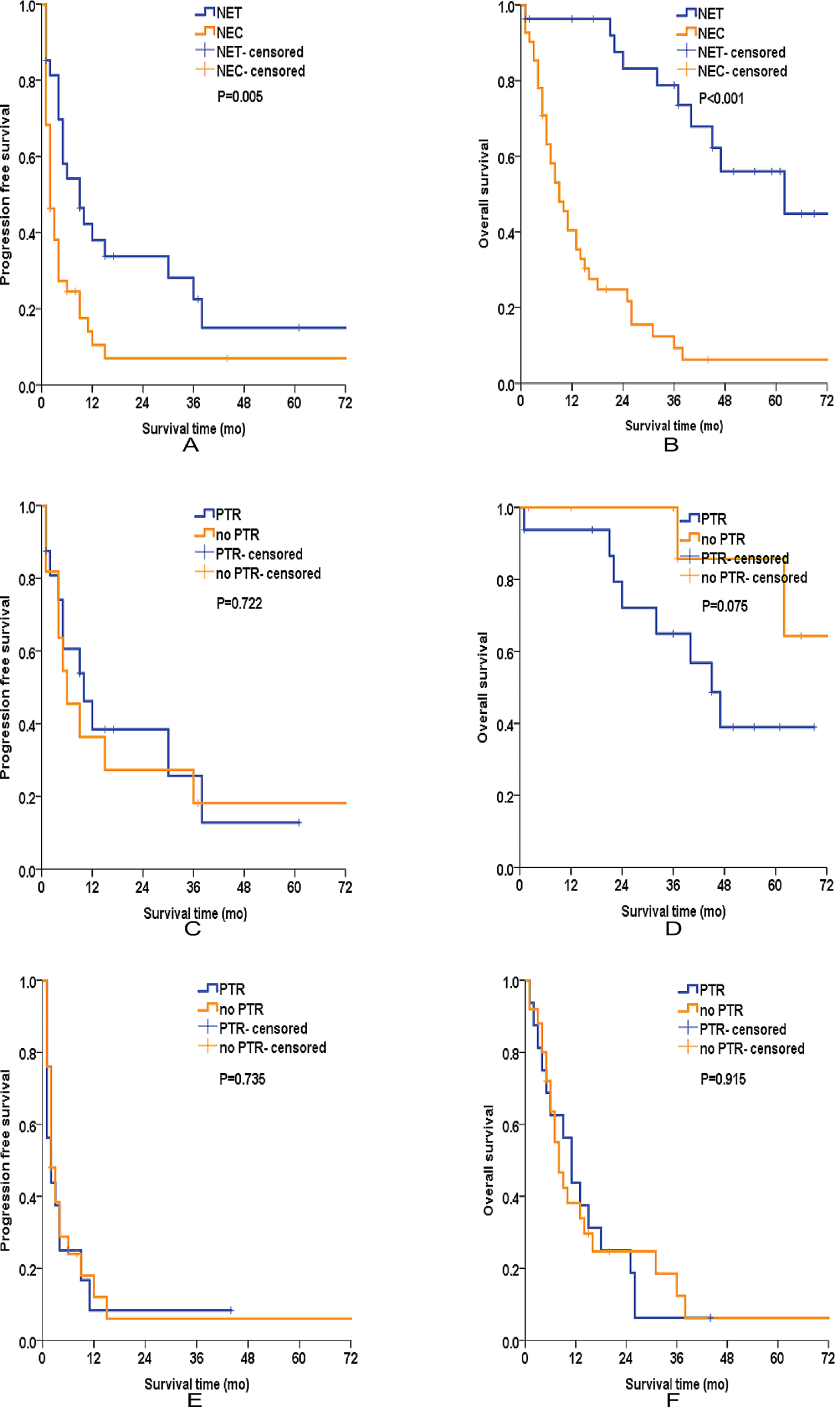
Kaplan–Meier survival analyses of patients after stratified by histological differentiation. (**A**) PFS of NETs and NECs in the whole cohort, (**B**) OS of NETs and NECs in the whole cohort, (**C**) PFS of patients who received PTR and those who did not in the NET subgroup, (**D**) OS of patients who received PTR and those who did not in the NET subgroup, (**E**) PFS of patients who received PTR and those who did not in the NEC subgroup, (**F**) OS of patients who received PTR and those who did not in the NEC subgroup. PTR: primary tumor resection, PFS: progression free survival, OS: overall survival

Given the histological differences between NET and NEC, we further performed subgroup analysis based on histological differentiation. In the NET group, the median PFS was 9 months, with 1-year and 3-year PFS rates of 38.0% and 22.5%, respectively. The median OS was 62 months, with 1-year and 3-year OS rates of 96.3% and 78.8%, respectively. The NEC subgroup had significantly worse PFS (*P* = 0.005) and OS (*P* < 0.001) than NET subgroup. In the NEC group, the median PFS was only 2 months, with 1-year and 3-year PFS rates of 10.5% and 7.0%, respectively. The median OS was only 9 months, with 1-year and 3-year OS rates of 40.4% and 9.3%, respectively (Fig. 2A and B). The Kaplan–Meier survival curves showed that there was no significant difference in terms of PFS and OS between the patients who underwent PTR and those who did not both in the NET subgroup (Fig. 2C and D) and the NEC subgroup (Fig. 2E and F).

### Univariable and multivariable analysis of prognostic factors

Univariable and multivariable Cox proportional hazards regression analyses were conducted in both the original cohort and the matched cohort to identify significant variables affecting PFS and OS (Tables 3 and 4). In the original cohort, the univariable and multivariable analysis indicated that histological differentiation [hazard ratio (HR) = 1.86, 95% confidence interval (CI): 1.02–3.41, *P* = 0.043] was the only prognostic factor for PFS. In terms of OS, univariable analysis showed that patients with primary colonic NENs (*P* = 0.011), G3 NENs (*P* < 0.001) and NECs (*P* < 0.001) had lower median OS than patients with primary rectal NENs, G1 and G2 NENs, and NETs. However, only histological differentiation was further verified as an independent risk factor for OS [HR = 3.70, 95% CI: 1.09–12.48, *P* = 0.035]. Palliative resection demonstrated no association with either PFS or OS in both the original cohort and the matched cohort.

**Table 3:** Univariable and multivariable Cox proportional hazards regression analysis for PFS and OS in the original cohort

Variables	PFS				OS			
	Univariable		Multivariable		Univariable		Multivariable	
	Median PFS (mo)	*P*	HR (95% CI)	*P*	Median OS (mo)	*P*	HR (95% CI)	*P*
Sex		0.385				0.980		
Male	3				24			
Female	4				18			
Age		0.251				0.128		
≤60	4				22			
>60	4				15			
Primary tumor site		0.056	1.37 (0.75, 2.51)	0.300		0.011	1.47 (0.67, 2.80)	0.241
Colon	5				31			
Rectum	2				9			
Grade		0.227				< 0.001	1.99 (0.50, 7.84)	0.326
G1 and G2	5				88			
G3	3				11			
Differentiation		0.012	1.86 (1.02, 3.41)	0.043		< 0.001	3.70 (1.09–12.48)	0.035
NET	9				62			
NEC	2				9			
Clinical T stage		0.724				0.745		
T1, T2	5				21			
T3, T4	4				22			
Clinical N stage		0.340				0.318		
N0	3				62			
N1	4				21			
Site of metastasis		0.861				0.310		
Single-organ	4				26			
Multiple-organ	4				18			
PTR		0.578				0.964		
Yes	4				24			
No	4				16			

PFS: progression free survival, OS: overall survival, HR: hazards ratio, CI: confidence interval, NET: neuroendocrine tumor, NEC: neuroendocrine carcinoma, PTR: primary tumor resection

**Table 4:** Univariable and multivariable Cox proportional hazards regression analysis for PFS and OS in the matched cohort

Variables	PFS				OS			
	Univariable		Multivariable		Univariable		Multivariable	
	Median PFS (mo)	*P*	HR (95% CI)	*P*	Median OS (mo)	*P*	HR (95% CI)	*P*
Sex		0.505				0.870		
Male	3				24			
Female	5				21			
Age		0.275				0.239		
≤60	4				24			
>60	3				25			
Primary tumor site		0.054	1.59 (0.80, 3.18)	0.186		0.077	1.31 (0.62, 2.74)	0.480
Colon	6				31			
Rectum	2				10			
Grade		0.437				< 0.001	1.06 (0.13, 8.82)	0.959
G1 and G2	5				62			
G3	3				10			
Differentiation		0.048	1.68 (0.85, 3.33)	0.136		< 0.001	5.14 (0.68–38.79)	0.112
NET	9				62			
NEC	2				9			
Clinical T stage		0.656				0.589		
T1, T2	9				62			
T3, T4	4				24			
Clinical N stage		0.873				0.263		
N0	5				62			
N1	4				24			
Site of metastasis		0.735				0.130		
Single-organ	4				32			
Multiple-organ	4				18			
PTR		0.613				0.983		
Yes	4				24			
No	4				31			

PFS: progression free survival, OS: overall survival, HR: hazards ratio, CI: confidence interval, NET: neuroendocrine tumor, NEC: neuroendocrine carcinoma, PTR: primary tumor resection

## Discussion

Based on the current results of clinical studies, systematic chemotherapy is the mainstay of treatment for patients with metastatic colorectal cancer (mCRC) and the most reliable choice to prolong OS [18]. Surgical resection with curative intent is only performed for patients with solitary metastasis, which account for only 10–15% of all mCRC [19]. For other mCRC, surgery is only indicated in dealing with emergent accidents of obstruction, bleeding and perforation. In recent years, many studies have explored the value of palliative PTR in improving survival. Although their results were controversial, most of these studies reported prolonged OS obtained from palliative PTR [20–23]. However, nearly all data concerning PTR of colorectal cancer were from studies of colorectal adenocarcinoma, as it is the main histological type of colorectal tumors. There remain few data exploring the survival benefits of palliative PTR for metastatic colorectal NENs.

Colorectal NENs are a group of heterogenous disease, their clinical manifestations and outcomes varied widely from G1 to G3 neoplasms. Although G1 and G2 NENs are regarded as well-differentiated and indolent disease with favorable prognosis, unresectable distant metastasis can still be found in some patients, even if the primary tumors are only small and T1 lesions [10]. For colorectal NENs of G3 grade and poor differentiation, over a half of patients presented with distant metastasis at the initial diagnosis, and most of these metastatic lesions cannot be resected with curable intent [9, 24].

Understanding how to prolong the OS of patients with metastatic colorectal NENs is thus an urgent issue. The median OS of all metastatic colorectal NENs were 24.8 months. However, for poorly differentiated NENs, the median OS dropped to only 8.7–10 months in literature reports [8, 9, 25]. In our study, it was 24 months in the entire cohort and 10 months for poorly differentiated G3 NENs, which was consistent with previous reports. Systematic chemotherapy and somatostatin analogs (SSA) have been the cornerstone in the treatment strategy of metastatic colorectal NENs based on the consensus guidelines from the European Neuroendocrine Tumor Society (ENETS) and North American Neuroendocrine Tumor Society (NANETS) [4, 26]. However, whether the primary colorectal NENs should be resected or not remains a controversial issue, and the efficacy of palliative surgery in improving survival has not been fully explored before. Previous reports have investigated the role of PTR of NENs of small bowel and pancreas. NANETs for small bowel NENs recommended to remove primary NENs to avoid future symptoms and have survival benefits [27]. Felix et al. performed one population-based study of 442 metastasized pancreatic NENs and demonstrated that palliative PTR was associated with significant improved survival [28]. Hua et al. extracted 1974 metastatic pancreatic NENs from Surveillance, Epidemiology, and End Results (SEER) database and concluded that palliative PTR can offer survival benefits [29]. Thus far, there was no reports specially targeting the role of PTR in only colorectal NENs through literature review. Most of previous studies included colorectal cases together with cases of the gastric, small intestine or pancreas, and explored the treatment efficacy in the whole group. Due to the heterogeneity of these reports, it is still unknown whether palliative PTR can offer survival benefits for patients with unresectable metastatic colorectal NENs. Strosberg et al. recruited 146 metastatic NENs of the mid-gut, most of which had the primary tumor located at the ileocecal region, they found no survival benefits obtained from PTR [30]. Lewis et al. included 854 metastatic gastrointestinal NENs, of which 81 cases were colorectal NENs, they reported improved OS offered by PTR independent of liver treatment and tumor grade [31]. Olatunji et al. reported 1861 poorly differentiated NECs based on the National Cancer Database (NCDB), 495 of them had the primary NECs in the large bowel, they demonstrated that surgical intervention of the primary tumor had been associated with favorable clinical outcomes [24]. Adam et al. also included 1208 colorectal NECs from NCDB, 405 of which present distant metastasis. They concluded surgical resection had offered better survival than those who had not, but whether these patients only received resection of the primary tumor, or both the primary and metastatic tumor was not detailed in this report [32]. However, Smith et al. reviewed 126 cases of colorectal NECs from a single American institution and found that resection of the primary tumor had no influence on OS for metastatic disease [8]. Additionally, another research from China argued against PTR for stage IV colorectal NECs [33].

To our knowledge, our study is thus far the first report specially focused on palliative PTR in colorectal NENs. Unlike prior reports that supported the decision of palliative PTR in the management of metastatic NENs, we didn’t observe a correlation between PTR and improved survival. PTR may not bring superior survival for these patients. Therefore, palliative PTR should be considered carefully to avoid delay of systematic chemotherapy, especially for asymptomatic cases. However, all current results were concluded from retrospective studies, prospective randomized control trials with more sample sizes still needed to explore the efficacy of PTR in improving survival. Moreover, we observed that tumor histological differentiation was the main prognostic factor affecting patients’ survival; colorectal poorly differentiated NECs presented significant worse prognosis than well-differentiated NETs. Given the significant difference in degree of malignancy, aggressiveness and clinical outcomes between NETs and NENs, it may be more reasonable to further explore the role of PTR in NETs and NECs patients alone in future reports.

Our study has several limitations. Firstly, it is retrospective in nature, as we included patients over a 20-year period, and thus, bias from patient selection and information collection cannot be entirely avoided. Secondly, although we collected patients from two Chinese medical centers, the sample size was still small, owing to the rarity of colorectal NENs. Thirdly, the majority of patients received PTR for relief of obstruction and bleeding, making it challenging to conclude whether PTR can offer a survival benefit for asymptomatic patients.

## Conclusions

In conclusion, palliative PTR may lack benefits for patients with unresectable metastatic colorectal NENs, the decision to do so should be made carefully for these patients, especially for asymptomatic patients.

## Data Availability

The datasets used during the current study are available from the corresponding author on reasonable request.

## Abbreviations

PTR: primary tumor resection
NEN: neuroendocrine neoplasms
PSM: propensity score matching
PFS: progression-free survival
OS: overall survival
SD: standard deviation
ECOG: Eastern Cooperative Oncology Group
NET: neuroendocrine tumors
NEC: neuroendocrine carcinomas
HR: hazard ratio
CI: confidence interval
mCRC: metastatic colorectal cancer
SSA: somatostatin analogs
ENETS: the European Neuroendocrine Tumor Society
NANETS: North American Neuroendocrine Tumor Society
SEER: Surveillance, Epidemiology and End Results
NCDB: National Cancer Database
